# Ointment of Iodine and Belladonna in Inflamed Mammæ

**Published:** 1858-09

**Authors:** J. S. Weatherly

**Affiliations:** Montgomery, Ala.


### ARTICLE III.

*Ointment of Iodine and Belladonna in Inflamed Mammæ.* By J. S. WEATHERLY, M. D., Montgomery, Ala.

The topical application of Iodine to the swollen and inflamed mammæ, has been a favorite treatment with the profession for sometime. Not, however, proving successful in all cases, and leaving room for some other remedy to be sought for. Recently Belladonna has been recommended quite highly as a successful remedy, not only for arresting inflammation of the mamma, but also of preventing the secretion of milk. Something to be very much desired in a great many cases. Having had several cases recently where it was very desirable to produce both of these effects, I concluded to try a combination of Iodine and Belladonna. I consequently applied an unguent, after the following formula: ℞. Iodine, grs. xx., ext. Belladonna grs. xx., cerate ℥i. Mix.

I have found this remedy to act admirably. The first case on which I used it, was a patient who had given birth to a still born child. Milk fever supervened with pain and swelling of the mammæ. The unguent was applied, affording almost instant relief. Nothing else was used and after the first day the patient suffered no more with her breast. Neither did she secrete any more milk. The second case was similar to the first, and was treated with a like result. I have used this combination frequently recently, always with good effect.

